# Muscle MRI at the time of questionable disease flares in Juvenile Dermatomyositis (JDM)

**DOI:** 10.1186/s12969-017-0154-4

**Published:** 2017-04-12

**Authors:** Rabheh Abdul-Aziz, Chack-Yung Yu, Brent Adler, Sharon Bout-Tabaku, Katherine E. Lintner, Melissa Moore-Clingenpeel, Charles H. Spencer

**Affiliations:** 1 Nationwide Children’s Hospital, 700 Children’s Dr, Columbus, OH 43205 USA; 2 Women & Children’s Hospital of Buffalo, 219 Bryant Street, Buffalo, NY 14222 USA

**Keywords:** MRI, Juvenile dermatomyositis

## Abstract

**Background:**

The course of JDM has improved substantially over the last 70 years with early and aggressive treatments. Yet it remains difficult to detect disease flares as symptoms may be mild; signs of rash and muscle weakness vary widely and are often equivocal; laboratory tests of muscle enzyme levels are often normal; electromyography and muscle biopsy are invasive. Alternative tools are needed to help decide if more aggressive treatment is needed. Our objective is to determine the effectiveness of muscle Magnetic Resonance Imaging (MRI) in detecting JDM flares, and how an MRI affects physician’s decision-making regarding treatment.

**Methods:**

This study was approved by the Institutional Review Board of Nationwide Children’s Hospital. JDM patients were consulted between 1/2005 and 6/2015. MRIs were performed on both lower extremities without contrast sequentially: axial T1, axial T2 fat saturation, axial and coronal inversion recovery, and axial diffusion weighted. The physician decision that a JDM patient was in a flare was considered the gold standard. MRI results were compared with physician’s decisions on whether a relapse had occurred, and if there was a concordance between the assessment methods.

**Results:**

Forty-five JDM patients were studied. Eighty percent had weakness at diagnosis, 100% typical rash, and 73% typical nail-fold capillary changes. At diagnosis, muscle enzymes were compatible with JDM generally (CK 52%, LDH 62%, aldolase 72%, AST 54% abnormal). EMG was abnormal in 3/8, muscle biopsy typical of JDM in 10/11, and MRI abnormal demonstrating myositis in 31/40. Thirteen patients had a repeat MRI for possible flares with differing indications. Three repeat MRI’s were abnormal, demonstrating myositis. There was moderate agreement about flares between MRI findings and physician’s treatment decisions (kappa = 0.59). In each abnormal MRI case the physician decided to increase treatment (100% probability for flares). MRI was negative for myositis in 10 patients, by which 7/10 the physicians chose to continue or to taper the medications (70% probability for non-flares).

**Conclusion:**

A muscle MRI would facilitate objective assessments of JDM flares. When an MRI shows myositis, physicians tend to treat 100% of the time. When an MRI shows no myositis, physicians continued the same medications or tapered medications 70% of the time. Further studies would help confirm the utility and cost-effectiveness of MRI to determine JDM flares.

**Electronic supplementary material:**

The online version of this article (doi:10.1186/s12969-017-0154-4) contains supplementary material, which is available to authorized users.

## Background

Juvenile dermatomyositis (JDM) is a chronic multisystem disease of presumed autoimmune origin. JDM is the most common of the idiopathic inflammatory myopathies of childhood, comprising 85% of cases [1]. It affects primarily skin and muscle, with less frequent involvement of other organs, including cardiovascular system, gastrointestinal tract, and lungs [2]. Although the outcomes of JDM have improved over the last few decades, the disease is still associated with significant morbidity [3].

The significant morbidity of JDM necessitates earlier treatment, earlier identification of flares and optimal treatment to prevent the associated morbidity.

Traditionally, the diagnosis of JDM was based on the Bohan and Peter criteria [4, 5]. Definite JDM consists of classic skin involvement and at least three of the following: 1) proximal muscle weakness, 2) elevation of muscle enzyme (s), 3) myopathic changes on electromyography (EMG) and 4) abnormal muscle biopsy suggestive of inflammatory myopathy [4, 5]. Probable JDM is defined as patients who have the characteristic rash and fulfill only two of the above criteria.

An expanded definition with more criteria was proposed in 2006 using an international consensus survey [2]. These new criteria include: 1) typical findings on muscle magnetic resonance imaging (MRI) and ultrasonography, 2) nailfold capillaroscopy abnormalities, 3) calcinosis, and 4) dysphonia [2].

Disease course was divided into three groups according to patterns of active and inactive disease: monocyclic, polycyclic, and chronic continuous, based on previous descriptions in the literature [6, 7]. One of the determining factors in patient response to treatment is whether the patient develops a disease flare after treatment is begun. In some patients it is difficult to determine a flare. JDM patients are usually treated with prednisone for one year according to Consensus Treatments for moderate JDM (CTP protocol) [8]. However, one possible side effect of prednisone is muscle weakness, which could confound detection of muscle weakness caused by flare during treatment. This means that deciding whether the new muscle weakness is due to JDM flare, side effects of steroids, or other causes may be a clinical puzzle. Usually in such cases we perform muscle enzymes assays, but results for tests may be normal despite a disease flare [9]. In these cases MRI results can help confirm the presence of muscle inflammation. Judicious use of MRI may offer the potential for further improving the management of JDM as it can aid in the assessment of occult active disease and muscle damage.

MRI is a non-invasive and well-tolerated examination. It is radiation free, painless, and can be repeated and compared with the initial MRI at diagnosis. MRI is preferred over EMG for evaluation and diagnosis of JDM. MRI is sensitive in detecting muscle inflammation, but it is not specific to a diagnosis of myositis because muscular dystrophies and other myopathies may have associated edema on MRI [2]. The signal changes on imaging need to be interpreted in the context of the clinical setting.

There is limited data about the role of MRI to determine disease flares when the clinical assessment and muscle enzymes are equivocal. Malattia et al., compared whole-body MRI (WB-MRI) with clinical examination to assess disease activity in 41 JDM patients at presentation, which they repeated in 18 patients at a median follow-up of 9 months [10]. They concluded that WB-MRI provides additional information to clinical evaluation and represents a promising tool to estimate total inflammatory burden, tailor treatment, and monitor its efficacy. Follow-up WB-MRI showed resolution of inflammation in nine patients whereas clinical criteria for remission were satisfied in five [10]. Our objective here is to determine if lower extremity muscle MRI assists in determining whether patients with JDM are in a disease flare. We hypothesize that MRI of the muscles may facilitate diagnose if a patient is having a disease flare when clinical assessment and laboratory markers are equivocal.

## Methods

This study protocol was approved by the Nationwide Children’s Hospital (NCH) Institutional Review Board (IRB). Written parental consent and patient assent were obtained. Patients were identified by diagnostic codes of Juvenile Dermatomyositis (ICD 10 code M33.90 and ICD9 code 710.3). Patients were included in the study if they met the modified Bohan and Peter criteria including MRI evidence of myositis (modification introduced by the Childhood Arthritis and Rheumatology Research Alliance (CARRA) Registry investigators) (2, 5), and characterized in a recent publication [11]. We included two JDM-like patients with predominant skin manifestations. Both patients have typical JDM rash and capillary dilatation, but never had any clinical muscle weakness. We included these patients as they could present with rash and then develop weakness later. We excluded patients with myositis in association with other connective tissue diseases, such as scleroderma and the overlap syndromes. A flare is considered when patients have a recurrence of active disease after a definite remission.

We analyzed the medical records retrospectively of children with JDM at NCH from January 2005 to June 2015. The data were collected using the NCH electronic medical record for the patients followed after 2007 and the paper chart for the patients followed from 2005 to 2007. Data were entered into an electronic database and were stored using linked anonymous codes. We recorded the following data: demographics, clinical presentation at the onset and at the time of repeat MRI, laboratory and images results, and therapeutics used. We specifically recorded the result of initial MRI and the corresponding muscle enzyme levels at the time of initial MRI. We also recorded the result of follow-up MRI and the corresponding muscle enzyme levels at the time. Additionally, we recorded the patient’s current treatment before and after the follow-up MRI. We looked at the sequence of following patients with flares including history, physical examination, levels of muscle enzymes, and we reviewed the physician assessment before and after the muscle MRI based on available chart information from the patient visits.

In our study, the MRI of both lower extremities was performed without gadolinium contrast utilizing the following sequences: axial T1, axial T2 fat saturation, axial and coronal inversion recovery, and axial diffusion weighted sequences.

Physician decisions to identify flares were considered the gold standard. Evidence of flare was noted by the presence of an active rash, a reduced childhood myositis assessment scale (CMAS) score for muscle weakness, and elevated levels of muscle enzymes after a remission lasting 6 months or more. We compared the MRI result for the presence of myositis or not with physician decisions and determined the concordance/discordance for flares or remission. Concordance occurred when MRI showed myositis and the physician’s decision was to treat the patient for a flare, either by increasing the current medications or by starting new medications. Concordance was also present if the MRI did not show active myositis and the physician did not treat the patient for a flare and continued the same medication, continued off medication if the patient was not on treatment, or tapered the current treatment. Cohen’s kappa was used to evaluate the chance-corrected agreement between physician decisions and MRI findings. Bayes’ rule was used to determine the conditional probability that the physician would decide to treat for flare or remission given the MRI findings. McNemar tests for paired proportions were also used to examine the agreement of MRI and elevated levels of muscle enzymes. All tests were 2-sided and p-values less than 0.05 were considered statistically significant.

## Results

Forty-five patients were identified and demographic and disease characteristics are shown in Tables 1 and 2.

**Table 1:** Demographic Features and Disease Characteristics of 45 Patients with Juvenile Dermatomyositis

Female/male	2.5: 1 (32 F/13 M)
Median age in years	5.8 (1.7–17.9)
Weakness	36/45 (80%)
Rash	45/45 (100%)
Nail fold capillary changes	33/45 (73%)
Calcinosis	5/45 (11%)
Monocyclic disease	12/39 (31%)
Polycyclic disease	12/39 (31%)
Chronic continuous	15/39 (38%)
Not classified (followed less than 2 years)	6

**Table 2:** Disease characteristics of 45 patients with juvenile dermatomyositis

Test name	Patients with completed test N (%)	Patients with abnormal results N (%)
CK	44/45 (98%)	23/44 (52%)
LDH	37/45 (82%)	23/37 (62%)
Aldolase	43/45 (95%)	31/43 (72%)
AST	44/45 (98%)	24/44 (54%
MRI at diagnosis	40/45 (89%)	31/40 (77%)
EMG	8/45 (18%)	3/8 (38%)
Muscle biopsy	11/45 (24%)	10/11 (90%)
Skin biopsy	2/45 (4%)	2/2 (100%)

**Fig. 1:**
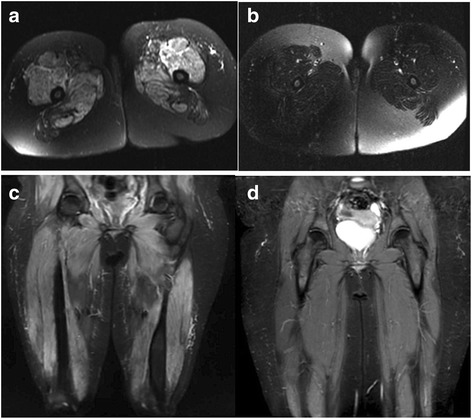
MRI at time of diagnosis and flares. **a** and **c** showed the axial and coronal section of the first MRI at the time of diagnosis. It showed diffusely increased T2 signal throughout all the muscles of the thigh as well as of the pelvis and hip girdle region with symmetrical involvement except for more patchy involvement in the adductors and in semi-membranous and semi-tendinous muscles. **b** and **d** showed the axial and coronal section of the second MRI at the time of flare. It showed: bilateral increased intramuscular T2 signal within both posterior calves including the lateral head of the gastrocnemius, soleus, and medial head of gastrocnemius and plantaris muscles with corresponding areas of restricted diffusion. The degree of restricted diffusion and T2 prolongation has markedly decreased compared with initial MRI A and C. There is subtle T2 prolongation with corresponding restricted diffusion diffusely within the anterior thigh musculature involving the bilateral vastus lateralis, rectus femoris and subtly within the vastus medialis muscles

The MRI was used at the initial presentation to determine the diagnosis of JDM in 40/45 patients. The repeat MRI was done in 13 patients. Two of the thirteen patients who had repeat MRI did not have an MRI performed at presentation. We stratified the patients with repeat MRI results according to the reason for repeating the MRI. All patients had history, physical examination, and muscle enzymes determined before the physician made a decision to follow up with MRI due to equivocal flare findings (Additional file 1). Reasons for repeating the MRI include thigh pain in one patient, calf pain in one patient (Fig. 1), hip pain in one patient, to determine remission in one patient, weakness in 4 patients, worsening nail fold capillary dilation without other signs of flare in one patient, rash in 3 patients, and an elevated muscle enzyme level without any other signs of flare in one patient.

MRI was performed in two patients (DM # 2 and DM # 7) for a third time. The reason for repeating the MRI again was to determine remission and whether or not to discontinue treatment. In DM # 2, the MRI showed no abnormality and a decision was made to continue tapering the medication. The patient did well without any flare thereafter. In DM # 7, the MRI showed bright T2 signal within the patellar tendons and superficial fasciae bilaterally. This patient continued on medications based on findings suggestive of active myositis in MRI. We did not include the third MRI in our data analysis to avoid bias.

Overall, there was moderate agreement between the second MRI findings and physician’s decision about flares (kappa = 0.519). However, based on conditional probability of treatment given MRI findings, the agreement is revealed to be more substantial. When the MRI findings show myositis, physicians tend to treat for myositis 100% of the time, while when MRI findings show no myositis, physicians tapered treatment 70% of the time.

As shown in Table 3, in two of the cases where MRI revealed myositis, the patients had no elevated enzymes. Conversely, in three of the patients with elevated enzymes, MRI did not show myositis. As expected, there was no statistically significant association between elevated muscle enzymes and MRI findings (McNemar’s test *p*-value = 1.0000) (Table 3).

**Table 3:** Association Between Elevated Muscle Enzymes and MRI Findings

	Myositis by MRI	No myositis by MRI	*p*-value (McNemar’s test)
Elevated enzyme(s)	1	3	1.0000
No elevated enzyme(s)	2	7	

## Discussion

In recent years, muscle magnetic resonance imaging (MRI) has played an increasingly important role in the diagnosis of inflammatory muscle disease. In many situations, MRIs have decreased the need for invasive procedures such as EMG and muscle biopsy [12, 13]. In our short report the muscle MRI was used in 89% at the time of diagnosis, while EMG and muscle biopsy were used in 17 and 24%, respectively. MRI could also be used in the selection of active disease sites for muscle biopsy as well [14]. In our study, we determined if the MRI assists in assessing whether patients with JDM are having a disease flare and how MRI might be affecting physician’s decision-making regarding treatment.

We showed that muscles MRI help decide if a patient is having a disease flare when the clinical assessment and laboratory markers are equivocal. When an MRI shows myositis at the time of questionable flares, physicians tend to treat 100% of the time and when an MRI shows no myositis, physicians in this study continued the same medications or tapered medications 70% of the time.

Previous studies showed that serum muscle enzyme values could be unreliable as markers for monitoring disease activity [15, 9]. Our study supported this conclusion as in 75% of the cases in which the MRI showed myositis, the patient had no abnormal enzymes. No statistically significant association were found between abnormal muscle enzymes and MRI findings (McNemar’s test p-value = 1.0).

There are limited previous studies on the use of the MRI to guide the decision of flares in JDM. Keim et al. reported the benefit of using the MRI at time of flare in one case [15]. Maillard et al. studied the finding of T2-weighted MRI scans of the thigh muscles in children with active JDM, inactive JDM and healthy children [16]. We showed the effective role of MRI in guiding the diagnosis of flares. Future direction of research may need to investigate this on a larger scale, study the cost effectiveness, and the role of MRI in definition of JDM remission. Disease remission is another questionable area and in many situations it is difficult to determine if the patient has an active or inactive JDM. The Pediatric Rheumatology International Trials Organization (PRINTO) established data-driven criteria with clear evidence-based, cut-off values to identify JDM patients with clinically inactive disease. The best combination of variables to classify a patient as being in a state of inactive disease on or off therapy were at least three of four of the following criteria: creatine kinase ≤150, Childhood Myositis Assessment Scale ≥48, manual muscle testing ≥78, and Myositis Disease Activity Assessment Visual Analogue Scale ≤0.2 [17]. Despite the use of these criteria, we still have some cases where it is difficult to define active or inactive disease similar to our 4-year-old patient (DM #6) where the judgment of CMAS was difficult due to her lack of cooperation with doing the CMAS evaluation. Including MRI findings may offer a better test to distinguish active or inactive JDM disease.

Our study should be interpreted in the light of its limitations; these include the retrospective nature of the study, the small sample size, and subjective definition of flares by using the physician's decision as a gold standard. In addition, we did not attempt to evaluate the cost effectiveness of an MRI in this clinical situation.

## Conclusion

Our study suggested that a muscle MRI might be valuable as a test to inform a physician’s decision about whether a child with JDM is having a disease flare when other findings or tests may not be helpful. There was a high concordance with physician decision making and the MRI. Further multicenter studies may be needed to confirm the utility and cost-effectiveness of MRI in detecting muscle flares in patients with JDM.

## Additional file

Additional file 1: Disease Characteristics of 45 Patients with Juvenile Dermatomyositis. (DOCX 27 kb)

## Abbreviations

AST: Aspartate aminotransferase
CARRA: Childhood Arthritis and Rheumatology Research Alliance
CK: Creatine kinase
CMAS: Childhood myositis assessment scale
CTP protocol: Consensus Treatments Protocol for moderate JDM
EMG: Electromyography
JDM: Juvenile dermatomyositis
LDH: Lactate dehydrogenase
MRI: Magnetic resonance imaging
NCH: Nationwide Children’s Hospital
PRINTO: Pediatric Rheumatology International Trials Organization
WB-MRI: Whole-body MRI
