# Supplementary material for: Muscle MRI at the time of questionable disease flares in Juvenile Dermatomyositis (JDM)
Source: Pediatr Rheumatol Online J. 2017 Apr 12;15:25. doi: 10.1186/s12969-017-0154-4 (PMC5389186; doi:10.1186/s12969-017-0154-4)
Supplement: Additional file 1: — Disease Characteristics of 45 Patients with Juvenile Dermatomyositis. (DOCX 27 kb) [file 12969_2017_154_MOESM1_ESM.docx]

| Concordance between MRI and treatment plan | Yes | Yes | Yes | No | No | Yes | Yes |
| --- | --- | --- | --- | --- | --- | --- | --- |
| Role of MRI | Helped in diagnosing JDM flare up and showed cellulitis. | Helped to determine flare up despite the normal muscle enzymes. | Helped in decision for escalating the treatment. | Did not help as it did not show myositis, but provider treat patient for flare up. | Did not help as no myositis, but provider treat patient for flare up. | Helped to rule out active myositis. | Helped to rule out myositis. |
| Treatment after MRI | Naficillin for cellulitis and IVIG for the JDM flare up | Continued on MTX and IVIG was started. | IVIG, prednisone. Methylprednisolone was increased to daily infusions for 3 days every month. mycophenolate mofetil was started | Started on methotrexate | MTX dose was increased | Continue weaning the patient’s medications. | Patient continued on same medications. |
| Treatment before MRI | Prednisone, mycophenolate mofetil, MTX*, weekly methylprednisolone | MTX | IVIG and methylprednisolone monthly, oral prednisone, and MTX. | MTX was discontinue 3 weeks before the MRI | MTX | prednisone with tapering and MTX | Prednisolone with tapering and MTX |
| MRI | Myositis and cellulitis | Myositis | Myositis | N | Hip effusion no myositis | N | N |
| AST | N | N | N | N | N |  | N |
| ALT | N | N | N | N | N | N | N |
| Aldolase | Elevated | N | N | Elevated | N/A | N | N |
| LDH | N | N | N | N | N/A** | N/A | N |
| CK | N | N | N | N | N | N | N |
| Reason for MRI | Right thigh pain, calcinosis over his thigh and fever. | Calf pain | Worsening weakness | Weakness and elevated Aldolase | Worsening of nailfold capillary dilatation while decreasing MTX | Determine of remission in a 4 year old with poor cooperation on exam | Rash |
| Patient | DM 1 | DM 2 | DM 3 | DM 4 | DM 5 | DM 6 | DM7 |

| Concordance between MRI and treatment plan | Yes | Yes | Yes | No | Yes | Yes | *MTX: methotrexate **N/A: not done  ***GBS: Guillaine Barre syndrome ****HCQ: Hydroxychloroquine |
| --- | --- | --- | --- | --- | --- | --- | --- |
| Role of MRI | Helped to rule out flare and to avoid starting new medications. | Helped rule out myositis and patient continued doing well. | Helped to rule out myositis. | Did not help as no myositis, but provider treated patient for flare. | Helped to avoid long term steroid use in the absence of active myositis. | Helped to rule out myositis. |  |
| Treatment after MRI | No new medication was started. | No medication | MTX and prednisone | started on prednisone | Steroid was started empirically given her weakness and then it was tapered as no myositis was detected on MRI. | HCQ****, then MTX was added for treating his rash without myositis at the time of flare. |  |
| Treatment before MRI | no medication | no medication | MTX and prednisone | MTX | Hydroxychloroquine and MTX | Hydroxychloroquine |  |
| MRI | N | N | N | N | N | N |  |
| AST | N | Elevated | N | N | N | N |  |
| ALT | N | N | N | N/A | N | N |  |
| Aldolase | N | N | N | N | N | N |  |
| LDH | N | N | N/A | N | N | N/A |  |
| CK | N | N | N | Elevated | N | N |  |
| Reason | Hip pain | Remission off of medication with no complaints and elevated AST | Rash | Weakness and elevated CK | Skin predominant JDM with weakness and GBS*** | Relapse after 11 years of first presentation of JDM with rash |  |
| Patient | DM 8 | DM 9 | DM 10 | DM 11 | DM 12 | DM 13 |  |
